# Mapping results for a set of cGAL effectors and drivers

**DOI:** 10.17912/W2Q947

**Published:** 2017-07-06

**Authors:** Sophie J. Walton, Han Wang, Jonathan Liu, Paul Sternberg

**Affiliations:** 1 Division of Biology and Biological Engineering, California Institute of Technology, Pasadena, California, USA; 2 Howard Hughes Medical Institute, California Institute of Technology, Pasadena, California, USA

## Description

Recently, the GAL4-UAS system (cGAL) has been adapted for use in *C. elegans* for control of gene expression across 15°C – 25°C (Wang et al., 2017). In order to create a desired gene expression pattern, one crosses a transgenic strain containing a driver construct with another strain containing an effector gene. Here we mapped several cGAL driver and effector integrations. We first crossed each of the cGAL driver and effector strains with N2 males, picked the heterozygous male progeny, crossed them with hermaphrodites of the mapping strain (DA438), picked L4 hermaphrodites with the corresponding transgenic marker of the driver or effector strain and scored the progeny in the next generation. The DA438 strain contains six recessive mutations, each of which locates on one of the six chromosomes and produces visible phenotypes (Bli on chromosome I, Rol on II, Vab on III, Unc on IV, Dpy on V, and Lon on X (Avery, 1993). F2 progeny with each of the six phenotypes were selected and examined for the presence or absence of the dominant marker associated with the transgene. In the cases where the dominant transgene marker is unlinked to the recessive phenotypic marker, about three quarters of the F2 progeny will have the dominant marker. If the two markers are linked, very few or no animals are expected to have the dominant transgenic marker. The following tables summarize the mapping results for each cGAL strain, stating the ratios of the F2 mutant progeny with and without the dominant transgenic marker.

**Effectors**

*15xUAS::GFP* for Cell Labeling

**Table:** 

Strain	Genotype	Location	Bli	Rol	Vab	Unc	Dpy	Lon
PS6843	syIs300	LGV	6:1	7:1	6:4	7:3	0:8	8:0
PS7149	syIs390	LGX	7:3	8:2	7:3	7:1	9:1	0:6

*15xUAS::mKate2* for cell Labeling

**Table:** 

Strain	Genotype	Location	Bli	Rol	Vab	Unc	Dpy	Lon
PS7136	syIs378	LGI	1:5	9:1	7:0	5:3	6:3	8:2

*15xUAS::hChR2(H134R)::YFP* for neuronal activation

**Table:** 

Strain	Genotype	Location	Bli	Rol	Vab	Unc	Dpy	Lon
PS7044	syIs341	LGIV	8:1	8:2	11:0	1:11	6:1	7:3
PS7045	syIs342	LGII	6:0	0:9	5:1	6:0	7:3	7:0

*15xUAS::TeTx* (Tetanus toxin light chain) for blocking synaptic transmission

**Table:** 

Strain	Genotype	Location	Bli	Rol	Vab	Unc	Dpy	Lon
PS7201	syIs421	LGIV	5:0	7:2	5:1	1:6	7:1	8:3

*15xUAS::HisCl1::SL2::GFP* for neuronal inhibition

**Table:** 

Strain	Genotype	Location	Bli	Rol	Vab	Unc	Dpy	Lon
PS7199	syIs371	LGIII	9:1	6:0	0:6	4:1	6:0	4:1
PS7107	syIs373	LGI	0:8	7:3	10:1	6:3	10:1	8:1
PS7108	syIs374	LGV	8:1	7:1	7:1	7:1	0:10	7:1

**Drivers**

Pharyngeal muscle driver, P*myo-2*

**Table:** 

Strain	Genotype	Location	Bli	Rol	Vab	Unc	Dpy	Lon
PS6844	syIs301	LGV	3:2	7:1	6:4	7:3	0:7	7:0

Heat shock driver, P*hsp-16.41*

**Table:** 

Strain	Genotype	Mapped	Location	Bli	Rol	Vab	Unc	Dpy	Lon
PS7169	syIs398 syIs337	syIs398	LGIII	8:0	7:0	1:4	7:0	7:0	6:0
PS7172	syIs401 syIs337	syIs401	LGIII	7:0	8:0	0:8	5:3	8:2	7:1

Pan-neuronal driver, P*rab-3*

**Table:** 

Strain	Genotype	Location	Bli	Rol	Vab	Unc	Dpy	Lon
PS6961	syIs334	LGX	6:1	5:1	6:1	5:1	8:2	2:7

Intestinal driver, P*nlp-40*

**Table:** 

Strain	Genotype	Location	Bli	Rol	Vab	Unc	Dpy	Lon
PS6935	syIs320	LGV	7:0	7:0	5:1	5:1	0:7	6:1

Body muscle driver, P*myo-3*

**Table:** 

Strain	Genotype	Location	Bli	Rol	Vab	Unc	Dpy	Lon
PS6936	syIs321	LGI	1:6	4:2	4:1	6:1	6:1	5:0

## Reagents

**Effector strains**

PS6843 *syIs300**[15xUAS::Δpes-10::GFP::unc-54 3’UTR + ttx-3p::RFP + pBlueScript]* V

PS7149 *syIs390**[15xUAS::Δpes-10::GFP::let-858 3’UTR + ttx-3p::RFP + 1kb DNA ladder(NEB)] X*

PS7136 *syIs378**[15xUAS::Δpes-10::mKate2::let-858 3’UTR + unc-122p::GFP + 1kb DNA ladder(NEB)]*I

PS7044 *syIs341**[15xUAS::Δpes-10::hChR2(Y134R)::YFP::let-858 3’UTR + ttx-3p::RFP + pBlueScript]* IV

PS7045 *syIs342**[15xUAS::Δpes-10::hChR2(Y134R)::YFP::let-858 3’UTR + ttx-3p::RFP + pBlueScript]* II

PS7201 *syIs421**[15xUAS::Δpes-10::TeTx::let-858 3’UTR + myo-2p::NLS::GFP + pBlueScript]* IV

PS7199 *syIs371**[15xUAS::Δpes-10::HisCl1::SL2::GFP::let-858 3’UTR + unc-122p::GFP + 1kb DNA ladder(NEB)]* III

PS7107 *syIs373**[15xUAS::Δpes-10::HisCl1::SL2::GFP::let-858 3’UTR + unc-122p::GFP + 1kb DNA ladder(NEB)]* I

PS7108 *syIs374**[15xUAS::Δpes-10::HisCl1::SL2::GFP::let-858 3’UTR + unc-122p::GFP + 1kb DNA ladder(NEB)]* V

**Driver strains**

PS6844 *syIs301**[myo-2p::NLS::GAL4SC::VP64::unc-54 3’UTR + unc-122p::RFP + 1kb DNA ladder (NEB)]* V

PS7169 *syIs398**[hsp16.41p::NLS::GAL4SK::VP64::let-858 3’UTR + unc-122p::RFP + 1kb DNA ladder(NEB)]* *syIs337**[15xUAS::Δpes-10::GFP::let-858 3’UTR + ttx-3p::RFP + 1kb DNA ladder(NEB)]* III

PS7172 *syIs401**[hsp16.41p::NLS::GAL4SK::VP64::let-858 3’UTR + unc-122p::RFP + 1kb DNA ladder(NEB)]* *syIs337**[15xUAS::Δpes-10::GFP::let-858 3’UTR + ttx-3p::RFP + 1kb DNA ladder(NEB)]* III

PS6961 *syIs334**[rab-3p::NLS::GAL4SK::VP64::let-858 3’UTR + unc-122p::RFP + pBlueScript]* X

PS6935 *syIs320**[nlp-40p::NLS::GAL4SK::VP64::unc-54 3’UTR + myo-2p::NLS::mCherry + pBlueScript]* V

PS6936 *syIs321**[myo-3p::NLS::GAL4SK::VP64::unc-54 3’UTR + myo-2p::NLS::mCherry + pBlueScript]* I

**Mapping strains**

DA438 *bli-4**(**e937**)* I*;* *rol-6**(**e187**)* II*;* *daf-2**(**e1368**)* *vab-7**(**e1562**)* III*;* *unc-31**(**e928**)* IV*;* *dpy-11**(**e224**)* V*;* *lon-2**(**e678**)* X

Wild type N2
